# New Insight into Secreted Ribonuclease Structure: Binase Is a Natural Dimer

**DOI:** 10.1371/journal.pone.0115818

**Published:** 2014-12-31

**Authors:** Elena Dudkina, Airat Kayumov, Vera Ulyanova, Olga Ilinskaya

**Affiliations:** Institute of Fundamental Medicine and Biology, Kazan Federal (Volga-Region) University, Kazan, Russia; Meharry Medical College, United States of America

## Abstract

The biological effects of ribonucleases (RNases), such as the control of the blood vessels growth, the toxicity towards tumour cells and antiviral activity, require a detailed explanation. One of the most intriguing properties of RNases which can contribute to their biological effects is the ability to form dimers, which facilitates efficient RNA hydrolysis and the evasion of ribonuclease inhibitor. Dimeric forms of microbial RNase binase secreted by *Bacillus pumilus* (former *B. intermedius*) have only been found in crystals to date. Our study is the first report directly confirming the existence of binase dimers in solution and under natural conditions of enzyme biosynthesis and secretion by bacilli. Using different variants of gel electrophoresis, immunoblotting, size-exclusion chromatography and mass-spectrometry, we revealed that binase is a stable natural dimer with high catalytic activity.

## Introduction

Ribonucleases (RNases) are enzymes that break down RNA and provide a necessary balance between the synthesis, operation and destruction of various RNAs. Degrading RNA serves multiple purposes within and outside cells. Cells must be cleared of RNA that is no longer needed and of RNA from foreign invaders. RNases secreted into the environment are non-specific, resulting in a very short lifespan for any RNA that is not protected. The function of these enzymes as critical epigenetic regulators provides signalling tools between cells [1]. Prokaryotic RNases in toxin-antitoxin systems are proposed to function as plasmid stability loci, and as stress-response elements when present on the chromosome [2], [3].

RNases possess a variety of biological activities if applied exogenously. Enzymes with an innate anticancer activity were revealed within both the RNase A and RNase T1 super-families. Binase, the small cationic guanyl-preferring RNase secreted by *Bacillus pumilus* (former *B. intermedius* [4]), was found to be cytotoxic and able to elicit selective apoptosis in cancer cells [5]–[9], as well as demonstrating antiviral activity against the pandemic influenza A (H1N1) virus [10].

Binase was sequenced by Aphanasenko et al. [11]. Its molecule contains three α-helices and two β-sheets [12]. The three-dimensional structure of binase was refined by Pavlovsky et al. [13] and Polyakow et al. [14]. In the crystal structures of binase, the characteristic dimer is present with the active site of one subunit being blocked owing to interactions within the dimer [8]. Since the dimers were found in crystals grown under four different conditions, it can be suggested that the enzyme exists as a dimer in solution as well [15]. Although only one native dimeric RNase, the bull semen RNase (BS-RNase), has been identified to date [16], RNase A can also form dimers under certain conditions [17]. Dimerisation is necessary for efficient RNA hydrolysis by RNase T from *E. coli* [18], for the specific decay of viral RNA by RNase L participating in innate immunity against microbial pathogens [19] and by monocyte chemoattractant protein 1-induced protein 1 (MCPIP1) [20], for the evasion of ribonuclease inhibitor RI by BS-RNase [21] and artificial dimers of RNase A [22]. We assumed that the ability of RNases to form dimers is a natural property that is more widespread than previously believed. The aim of this study was to find experimental proof confirming the existence of binase in a native dimeric form under natural conditions. Here, we have shown that binase formed stable dimers in solution. Moreover, it is secreted by *B. pumilus* as a highly catalytically active dimer. This finding will contribute to the elucidation of the precise molecular mechanism of the anti-tumour and antiviral effects of RNases, which still remain unclear.

## Materials and Methods

### Bacteria and growth conditions

*Bacillus pumilus* (former *B. intermedius,* strain number B-3073 in the Russian Collection of Microorganisms, Genetics Institute, Moscow, Russia) was used as a source of binase (EC 3.1.27.3; single chain of 109 amino acids, molecular weight 12.3 kDa). *E. coli* JM107 pML163 was used for recombinant binase purification [23]. Apart from the structural gene of *birA* (Binase), the plasmid pML163 is identical to the barnase expression plasmid pMT416 [24], with the enzyme on a *tac* promoter and *E. coli phoA* signal sequence and with the inhibitor barstar under the control of its own promoter [25].

*B. pumilus* was grown on the complex phosphate deficient LP medium (low phosphate peptone, 2.0%; glucose, 1.0%; CaCl_2_, 0.01%; MgSO_4_×7H_2_O, 0.03%; NaCl, 0.3%; MnSO_4_, 0.01%; pH 8.5). *E. coli* was grown on LB medium supplemented with ampicillin (100 µg/ml) for recombinant strains.

### Enzyme preparation

Binase was isolated from the medium of both *B. pumilus* (wild-type enzyme designated as “native” binase) and *E. coli* JM107 pML163 (recombinant binase), as described previously [26], [27]. The additional purification of binase was carried out using the MonoS column (HR 10/10, Sigma), equilibrated with 20 mM Na acetate buffer (pH 5.0). Proteins were eluted using a linear gradient of 0–0.25 M NaCl and lyophilised. This method is well-known and gives a pure binase sample, which has been repeatedly verified by different methods [26], [27].

### Catalytic activity

The catalytic activity of binase was 1.4×10^7^ U/mg when measured against high molecular weight yeast RNA [28]. One unit was defined as the amount of enzyme that increases the extinction of acid-soluble products of RNA hydrolysis at 260 nm per min at 37°C. The activity was measured in buffer containing 250 mM Tris-HCl, pH 8.5.

In-gel RNase activity measurements were performed as zymography of protein samples separated in 15% polyacrylamide gel with 0.1% SDS (SDS-PAGE) [29]. For that, the resolving gel was supplemented with 7 mg/mL RNA from Torula yeast (Sigma-Aldrich, USA) prior to polymerisation. After electrophoresis, the gel was washed with buffer I (10 mM Tris-HCl, 20% isopropanol, pH 7.5) for 10 min to remove SDS and then proteins were refolded by consequent incubation for 10 min in 10 mM Tris-HCl, pH 7.5 and in 100 mM Tris-HCl, pH 7.5. The gel was stained for 10 min with 0.2% toluidine blue (Sigma-Aldrich, USA).

### Mass spectrometry

Proteins were excised from the Coomassie-stained PAGE as 1.5×1.5 mm fragments. After washing with acetonitrile and 200 mM NH_4_HCO_3_ (mixed in ratio of 1∶1) to remove Coomassie R250 from the samples, proteins were digested with trypsin (Promega) overnight at 37°C. The resulting peptides were extracted using 0.1% trifluoroacetic acid and dried in a vacuum concentrator. LC-MS/MS analyses were performed on an HPLC LC-MS/MS system (Bruker, Germany).

### Cross-linking reactions with glutaraldehyde

To analyse the dimer formation by binase in solution, cross-linking experiments with glutaraldehyde were performed as described in [30]. Briefly, proteins (0.1 µg/µl) were pre-incubated in buffer (10 mM K_2_HPO_4_, 100 mM NaCl, 2 mM MgCl_2_, pH 7.4) at 4°C for 10 min. Then, the glutaraldehyde was added until a final concentration of 0.1%, followed by incubation for 10 min at 25°C. The reaction was stopped by the addition of Tris-HCl pH 7.4 until a final concentration of 25 mM. The samples were analysed by SDS-PAGE.

### Protein separation by gel electrophoresis and immunoblotting

To estimate the purity and molecular weight of binase samples, proteins were separated by SDS-PAGE [29]. After electrophoresis, the proteins were transferred to a nitrocellulose membrane by semi-dry electroblotting. Anti-binase antibodies were isolated from rabbit blood as described earlier [31]. Antibodies were visualised using anti-rabbit IgG-POD secondary antibodies (Sigma) and the LumiLight detection system (Roche Diagnostics).

To assess dimer formation by binase, native PAGE was performed under acidic conditions without any denaturing agents, using the modified Laemmli's protocol [29]. Stacking (15% acryl amide) and separating (5% acryl amide) gels contained 1.5 M Acetate-KOH (pH 4.8) and 0.25 M Acetate-KOH (pH 6.8), respectively. Protein samples were suspended in an equal volume of sample buffer (0.25 M Acetate-KOH, 10% glycerol, 0.02% methyl green, pH 6.8). Electrophoresis was performed using the following conditions: 1 mA/cm for the stacking gels and 2 mA/cm for the separating gels. The gels were for 1.5 h to 2 h in running buffer (0.35 M β-alanine, 0.14 M Acetate-KOH) and then stained with Coomassie R250 (Sigma-Aldrich, USA).

### Size-exclusion chromatography

The samples were separated using Superdex 200 (10/300 GL, Sigma) columns at a flow rate of 0.5 ml/min in buffer A (50 mM Tris-HCl, 100 mM KCl pH 8.0) on a Waters breeze HPLC system, USA. Molecular weights of binase peaks were calculated using the equation obtained from the elution profile of marker proteins with known molecular weights (bovine serum albumin (BSA), MW 66.2 kDa; trypsin, MW 24 kDa; papain, MW 23.7 kDa; lysozyme, MW 14.4 kDa) (Sigma-Aldrich, USA).

## Results

### Binase forms stable dimers in solution

**Figure 1:**
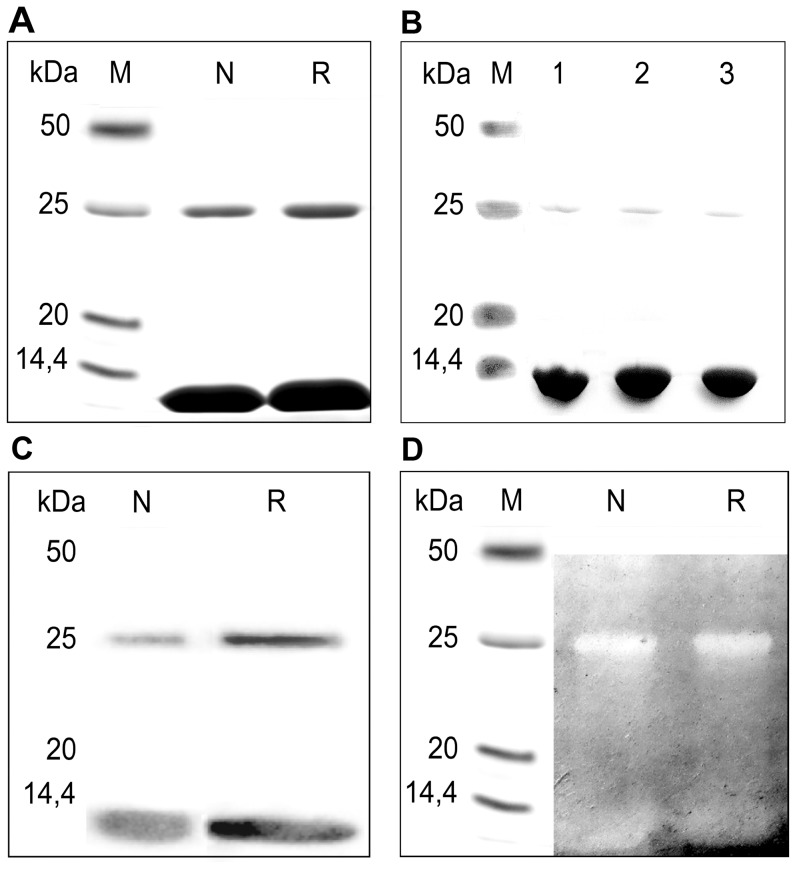
Identification of binase dimers. (A) The SDS-PAGE in presence of 0.1% SDS, (B) SDS-PAGE of native binase under strong denaturing conditions (1–2% SDS, 2–4 M urea, 3–8 M urea), (C) immunoblot detection and (D) in gel RNase activity assay of lyophilised binase from wild-type *B. pumilis* 7P (native, N), *E. coli* JM107 (recombinant, R).

In previous works, binase was considered to be a monomer with a molecular weight of 12.3 kDa [27]. Here, we purified wild-type and recombinant binase samples from *B. pumilus* 7P and *E. coli* JM107 pML163; in both cases, two bands with molecular weights of 12 and 25 kDa appeared after SDS-PAGE (Fig. 1A). A two-fold increase of SDS concentration in the sample buffer or addition of the strong denaturing agent urea led to the disappearance of the 25 kDa band, but not completely (Fig. 1B). Furthermore, both bands were able to interact with anti-binase antibodies in the immunoblot analysis (Fig. 1C). To characterise these two bands, an in-gel RNase activity assay was performed (Fig. 1D). Here, the binase samples were separated by SDS-PAGE and refolded, as described in the Materials and Methods section. Surprisingly, both bands showed RNase activity, confirming the presence of RNase in the upper band. It should be noted that both the wild-type and recombinant binase demonstrated the same SDS-PAGE pattern.

These facts allowed the suggestion that the protein of the upper band could be a dimeric form of binase. To check for the presence of another RNase or some unknown protein that may be coupled with binase in the 25 kDa band, we carried out the tryptic hydrolysis of both bands followed by mass spectrometry analysis performed on the HPLC LC-MS/MS system. According to the spectrometry data, both proteins were determined as binase (*B. intermedius* RNase), with protein sequence coverage of 85% (S1 Table). No other proteins were identified, confirming that the 25 kDa band represents the dimeric form of binase. On the basis of protein band emission in the gel (Fig. 1A) and the size of the corresponding RNA digestion zones (Fig. 1D), an approximate evaluation of RNase activity ratio to protein content obtained using BioRad ImageZone software showed, that protein amount of low molecular band was 4.5-fold higher than of high molecular band, and the size of corresponding RNA digestion zones of 25 kDa band was 1.7-fold higher than of 12 kDa. Finally, the specific activity of 25 kDa band was roughly 8-fold higher than that of 12 kDa band.

### Binase is a fully dimeric protein in solution

**Figure 2:**
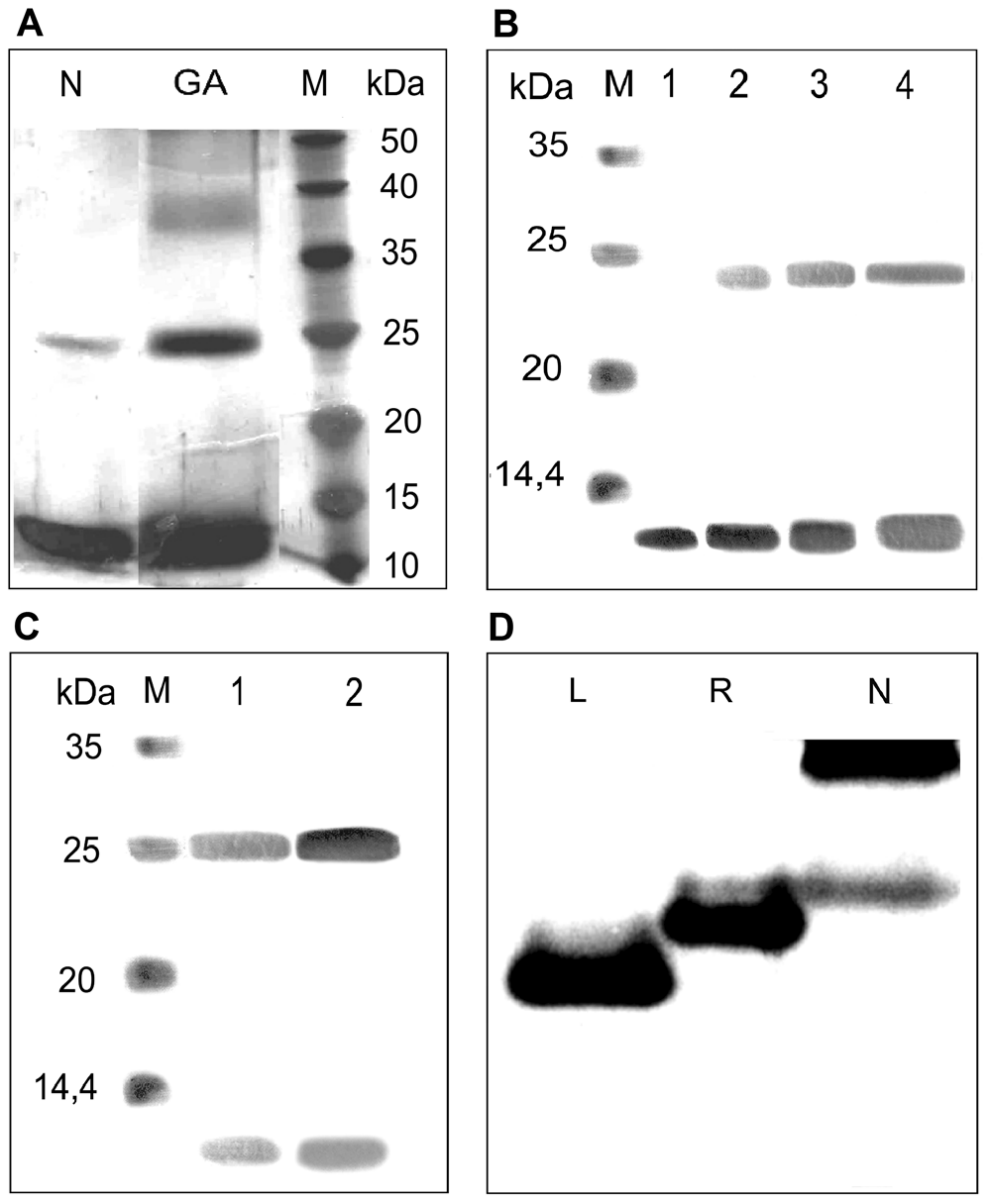
The evidences of binase dimerisation. (A) The cross-linking analysis of native binase (native, N) and treated by glutaraldehyde (GA) at final concentration 0.5 mg/ml and at increasing protein concentrations: (B) native binase, (C) cross-linked with GA (1–0.05 mg/ml, 2–0.1 mg/ml, 3–0.5 mg/ml, 4–1 mg/ml), (D) native-PAGE of native binase (L – lysozyme (MW  = 14.4 kDa, pI 11.3), R – RNase A (MW  = 13.7 kDa, pI 9.64), N – binase (MW  = 12 and 25 kDa, pI 9.5).

We have shown that binase exists on SDS-PAGE in both its monomeric and dimeric forms (Fig. 1A), which fits with previous data showing that binase forms dimers in a crystal and probably in solutions with high protein concentrations [8], [15]. The question is addressed to the ratio of binase monomers and dimers in solution in the absence of denaturing agents. To generate the reference form of a dimeric binase, the cross-linking of binase by glutaraldehyde was performed. The cross-linked dimer showed a thick band with the same molecular weight of 25 kDa, confirming the chemical dimerisation of binase (Fig. 2A). Nevertheless, a certain amount of monomer was also identified. It is possible to refer the monomer to the incomplete cross-linking and denaturation of dimeric form, which was not stabilised by glutaraldehyde. To disprove the hypothesis that binase only forms dimers at high concentrations, this analysis was performed using different concentrations of the enzyme (0.1, 0.5 and 1 mg/ml). The dimer band was detected at all concentrations used, except for the smallest one (Fig. 2B). However, dimer stabilisation by glutaraldehyde revealed the presence of dimer in 0.1 mg/ml binase sample as well (Fig. 2C). The electrophoresis under non-denaturing conditions without SDS revealed the significant prevalence of the dimer (Fig. 2D) with a weak band corresponding to the binase monomer.

**Figure 3:**
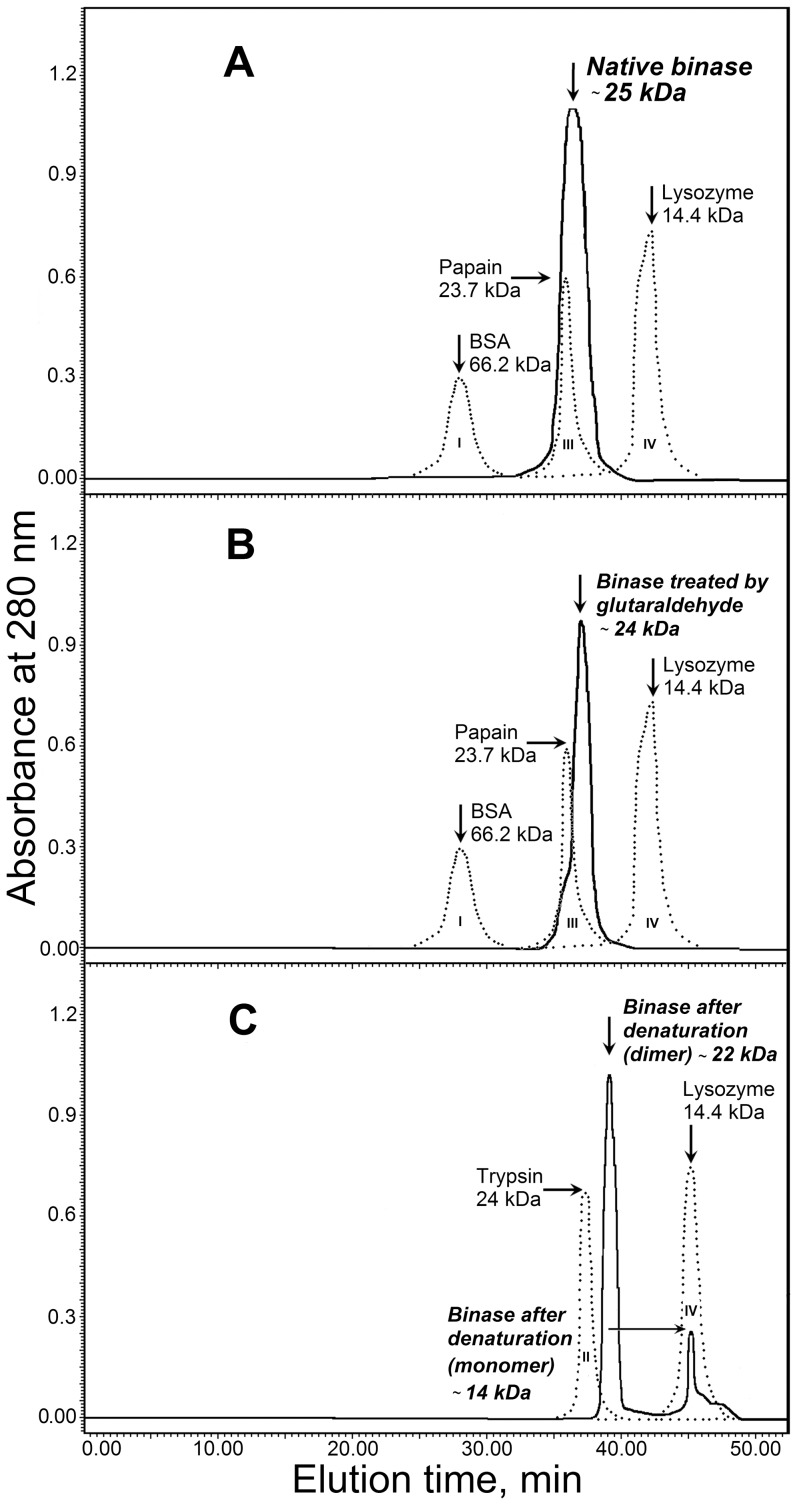
Size exclusion chromatography of binase at pH 8.0 (A, B) and pH 3.0 (C). A –native binase; B – binase after cross-linking by glutaraldehyde; C - binase denatured by heating for 20 min at 95°C, pH 2.5; I–IV – marker proteins used for molecular weight estimation: BSA (66.2 kDa), trypsin (24 kDa), papain (23.7 kDa), lysozyme (14.4 kDa) correspondingly. Molecular weights of binase peaks were calculated using equation obtained from elution profile of marker proteins.

Finally, binase dimerisation was studied by size-exclusion chromatography using the Superdex 200 column (Fig. 3). For the native binase, only one peak with a molecular weight of 25 kDa was identified (Fig. 3A). The same elution profile was obtained if the glutaraldehyde-treated binase was loaded (Fig. 3B). The specific activity of fractions collected between 35 and 40 min was similar to the activity of the protein sample loaded onto the column. Measured catalytic activity was 1.4×10^7^ U/mg. No activity was detected in other fractions, confirming that, under native conditions, binase exists as the fully dimeric form. Additionally, the protein was denatured by heating at 95°C for 20 min under acidic conditions (pH 2.5) and chromatography was carried out in acidic buffer (pH 3.0) (Fig. 3C). Here, together with a 22-kDa peak, a small protein peak with a calculated molecular weight of 14 kDa was detected. This demonstrates the partial dissociation of dimers. Fractions corresponding to both peaks were collected and transferred to the buffer with pH 8.5. After 30 min renaturation, the ribonucleolytic activity was detected, confirming the RNase nature of both peaks. These facts allow us to conclude that binase easily dimerises even after denaturation-renaturation cycle, thus retaining its activity, and is present only as a dimer in solution.

### Binase dimerisation in vivo

Based on our results confirming the dimeric state of binase in lyophilised samples of pure enzyme, we assumed that the binase secreted by *B. pumilus* into the medium is a dimer. To check this assumption, *B. pumilus* was grown on LP-medium, samples of the culture fluid were collected after 12 and 24 hours of growth (exponential and stationary phases), and binase was detected in the samples by immunoblotting after separation by SDS-PAGE.

**Figure 4:**
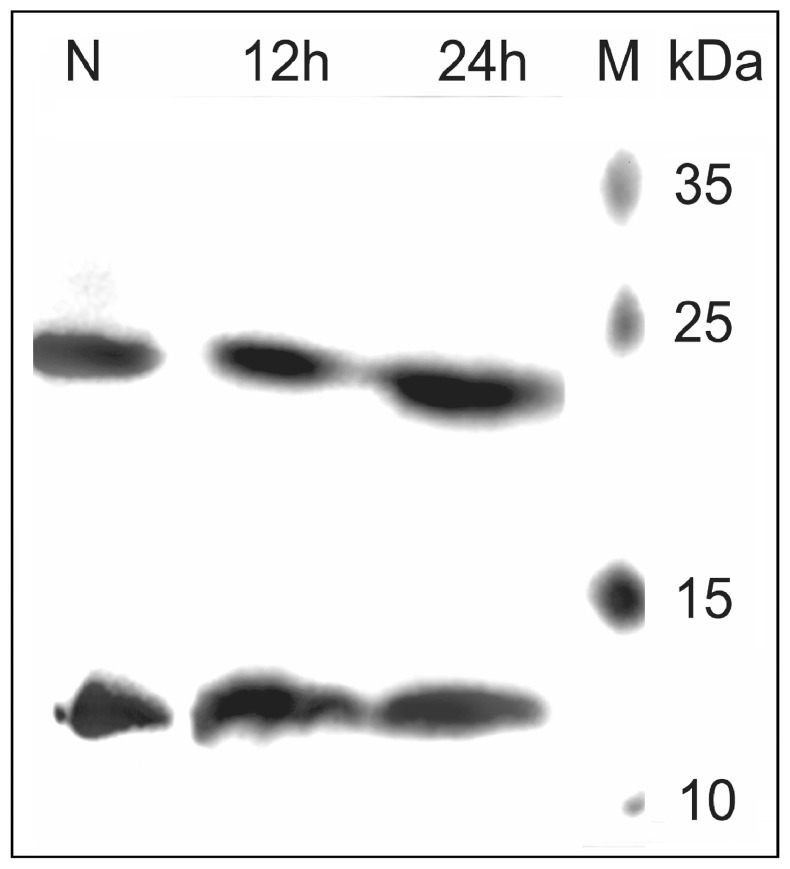
Western Blot analysis of *B. pumilis* 7P culture fluid collected after 12 h (exponential phase) and 24 h (stationary phase) of cultivation. N- lyophilised binase from wild-type *B. pumilis* 7P.

The protein amount was normalised by RNase activity since less binase was present in the medium on the exponential phase. As a control, the lyophilised binase was applied (Fig. 4). The binase dimer was identified in both medium samples harvested following 12 and 24 hours of growth, confirming that binase is a stable native dimer.

## Discussion

Almost all known bacteria secrete extracellular RNases. *Bacilli* produce at least 2 types of RNases, the “high-molecular weight” RNases [32] and small guanyl-specific RNases, among which binase, the thermostable enzyme from *B. pumilus* (originally named *B. intermedius*), and barnase from *B. amyloliquefaciens* are the best characterised [4], [11], [25], [27]. Despite classical reports describing the monomeric form of binase [11], [27], many studies have demonstrated the dimerisation of binase in crystals [8], [13], [14], [33]. Actually, the ability to form dimers is not surprising for RNases and was shown to endow them with additional biological properties such as allostery, anti-tumour and immunosuppressive activity, improved stability and control over the accessibility and specificity of active sites [17], [34], [35].

Here, we show for the first time that binase is almost completely found in the dimer form in both solution (Fig. 2D, Fig. 3) and in the culture fluid of *B. pumilus* (Fig. 4). The dimerisation does not depend on the concentration of the protein (Fig. 2B, Fig. 2C) as suggested previously [8], [15]. Moreover, binase is quite stable: it does not dissociate completely even in the presence of 2% SDS or 8 M urea (Fig. 1B) and easily redimerises after denaturation (Fig. 3C). The absence of sulfhydryl groups in the binase molecule excludes the potential for covalent oligomerisation, which was established for naturally homodimeric BS-RNase linked by two intermolecular disulfide bonds [36]. Monomeric ribonuclease A lyophilised from 50% acetic acid is also able to form dimers via swapping its C- and, to a lesser extent, N-termini [37]. Interestingly, disulfide bonds between the two subunits are absent, while H bonds play a significant role [38], [39].

For sulfur-free proteins like binase electrostatic interactions, the hydrophobic effect and H-bonds should play an important role in subunit association. Polyakov et al. [14] have reported that binase forms non-covalent dimers in crystal, which are stabilised by hydrogen bonds and hydrophobic interactions. Hydrophobic residues (except Ala) and the charged residue Arg are predominantly present at protein-protein interfaces with Tyr and Trp having the highest propensity [40]. Shirshikov et al. [41] proposed that the hydrophobic region of α-helix II on the N-terminus participates in the interaction between binase monomers with Leu-32 being especially important.

According to our size-exclusion chromatography data, binase is almost fully dimerised, possessing high catalytic activity (Fig. 3). Taking into account the higher catalytic activity of dimers per protein amount in the in-gel activity assay compared with monomers (Fig. 1A, D), we propose that both catalytic centres of the dimer participate in catalysis. The main residues of the active site identified in structural complexes from binase with nucleotide-type ligands (with bases: Glu59, Phe55, and with phosphates: Glu72, Arg86 and His101) [42] are available for substrate binding in both subunits. This assumption could be confirmed by the results obtained by the Brownian dynamics simulation method [43]. It was shown that three types of binase dimers could be formed. The first type leaves the active centre of binase free. In this dimer, residues Arg15, Arg107 and Arg109 from one monomer are in a close contact with residues Asp11 and Asp7 from the second monomer. An association rate constant of the type I dimer is the largest one among all three types and is comparable to that of binase and barstar inhibitor [43], forming tight complex stabilised by electrostatic interactions [44]. In dimer types II and III, the active centre of one or both binase molecules is blocked, which makes the enzyme partially or fully inactive [43]. This reflects the dimer state in crystals, where the active site of one subunit of binase is blocked owing to interactions within the dimer [8].

**Figure 5:**
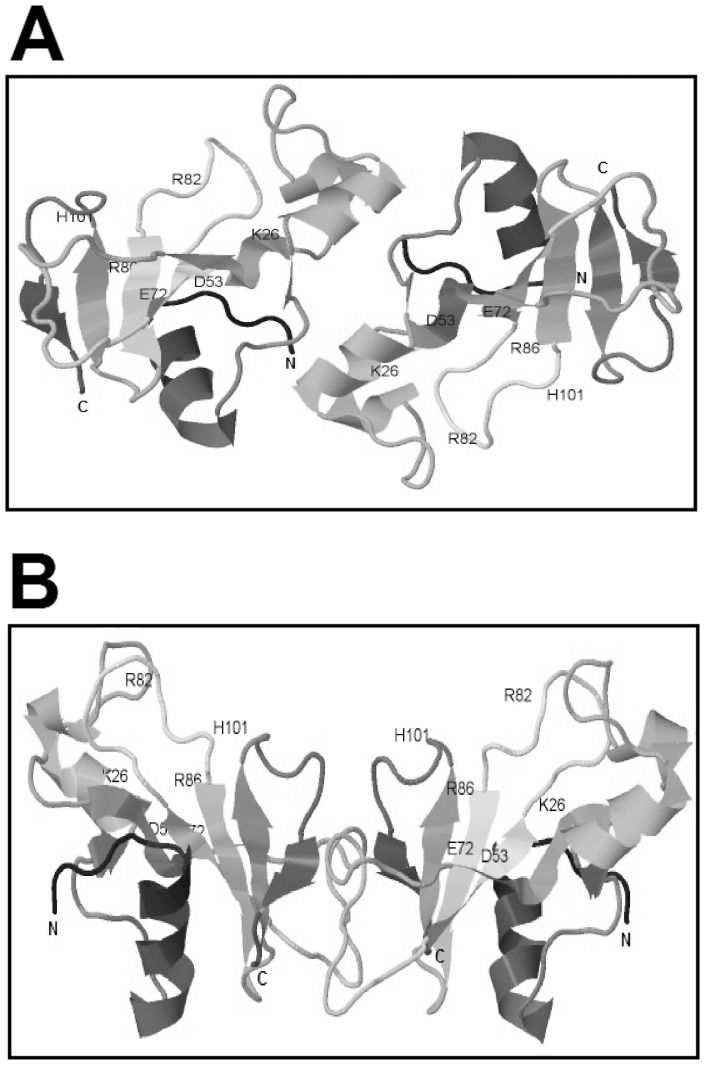
Putative models of binase dimer in solution. (A) The model of van der Waals and electrostatic-favored cluster. (B) The model of balanced and electrostatic-favored clusters. Amino acid residues constituting the active centre of enzyme are labelled. The models were generated using atomic coordinates of binase (PDB 1buj) on ClusPro server [38]. Visualisation was performed by Jmol: an open-source Java viewer for chemical structures in 3D (http://www.jmol.org/).

We also performed modelling of binase dimers based on the protein structure in solution (PDB 1BUJ) using ClusPro [45]. The algorithm implies 70000 rotations of one molecule around another, with 1000 rotations having the lowest energy score, which are further clustered with a 9 angstrom C-alpha rmsd radius and ranked according to the cluster size. Based on the cluster size criteria and taking into account the availability of active centres to the substrate and putative involvement of the hydrophobic core in inter-subunit interactions, we selected two putative models of the binase dimer. The first model (Fig. 5A) is based on electrostatic interactions and van der Waals contacts; the hydrophobic segment suggested by Shirshikov et al. [41] is also involved in the interaction. The second model (Fig. 5B) reflects a balance of binding forces with the predominance of electrostatic interactions. Both models represent a good basis for 3D N-terminal (Fig. 5B) and C-terminal (Fig. 5A) domain swapping, which is the dimerisation mechanism known for many proteins, including BS-RNase and RNase A [37], [46]. BS-RNase exists as a mixture of swapped and unswapped dimers in a 7∶3 ratio [47]. For RNase A, major C-terminally and minor N-terminally swapped dimers are described [38]. We propose that binase also exists in dimeric conformations differing by stability, because the partial dimer destruction was registered in all types of SDS-PAGE (Figs. 1 and 2). Nevertheless there is no doubt that binase is a natural dimer and all its biological properties, such as antitumor and antiviral activities refer to dimeric form of enzyme.

## Supporting Information

S1 Table **Identification of proteins in SDS-PAGE bands by mass spectrometry using peptide mass fingerprinting.**
(DOCX)
