# Supplementary material for: New Insight into Secreted Ribonuclease Structure: Binase Is a Natural Dimer
Source: PLoS One. 2014 Dec 31;9(12):e115818. doi: 10.1371/journal.pone.0115818 (PMC4281067; doi:10.1371/journal.pone.0115818)
Supplement: S1 Table — Identification of proteins in SDS-PAGE bands by mass spectrometry using peptide mass fingerprinting. (DOCX) [file pone.0115818.s001.docx]

Supplementary materials

Table S1. Identification of proteins in SDS-PAGE bands by mass spectrometry using peptide mass fingerprinting.

Table S1

| Binase sample | Taxonomy /Identification results | MM, Da | pI | Protein sequence coverage | Protein sequence |
| --- | --- | --- | --- | --- | --- |
| Binase monomer | Firmicutes (gram-positive bacteria) /*Bacillus intermedius*/ chain A, hydrolase (endoribonuclease) ribonuclease Bi (G specific endonuclease) (E.C.3.1.27.) complexed with guanosine-3'- phosphate (3'-Gmp) | 12 950 | 9.47 | 90 | FTPVTKAAVINTFD  GVADYLIRYKRLPN  DYITKSQASALGWV  ASKGDLAEVAPGKSI  GGDVFSNREGRLPS  AGSRTWREADINYV  SGFRNADRLVYSSD  WLIYKTTDHYATFTRIR |
| Binase dimer | Firmicutes (gram-positive bacteria) /*Bacillus intermedius*/ chain A, hydrolase (endoribonuclease) ribonuclease Bi (G specific endonuclease) (E.C.3.1.27.) complexed with guanosine-3'- phosphate (3'-Gmp) | 12 950 | 9.47 | 87 | FTPVTKAAVINTFDG  VADYLIRYKRLPND  YITKSQASALGWVA  SKGDLAEVAPGKSIG  GVFSNREGRLPSAGSR  TWREADINYVSGFR  NADRLVYSSDWLIY  KTTDHYATFTRIR |
